# The Vulnerability of Vessels Involved in the Role of Embolism and Hypoperfusion in the Mechanisms of Ischemic Cerebrovascular Diseases

**DOI:** 10.1155/2016/8531958

**Published:** 2016-05-29

**Authors:** Yong Peng Yu, Lan Tan

**Affiliations:** ^1^Department of Neurology and Central Laboratory of Brain Diseases, Weihai Central Hospital, School of Medicine, Qingdao University, Shandong 264400, China; ^2^Department of Neurology, Qingdao Municipal Hospital, School of Medicine, Qingdao University, No. 5 Donghai Middle Road, Qingdao 266071, China

## Abstract

Accurate definition and better understanding of the mechanisms of stroke are crucial as this will guide the effective care and therapy. In this paper, we review the previous basic and clinical researches on the causes or mechanisms of ischemic cerebrovascular diseases (ICVD) and interpret the correlation between embolism and hypoperfusion based on vascular stenosis and arterial intimal lesions. It was suggested that if there is no embolus (dynamic or in situ emboli), there might be no cerebral infarction. Three kinds of different clinical outcomes of TIA were theoretically interpreted based on its mechanisms. We suppose that there is a correlation between embolism and hypoperfusion, and which mechanisms (hypoperfusion or hypoperfusion induced microemboli) playing the dominant role in each type of ICVD depends on the unique background of arterial intimal lesions (the vulnerability of vessels). That is to say, the vulnerability of vessels is involved in the role of embolism and hypoperfusion in the mechanisms of ischemic cerebrovascular diseases. This inference might enrich and provide better understandings for the underlying etiologies of ischemic cerebrovascular events.

## 1. Introduction

As is generally known, ischemic cerebrovascular disease (ICVD) is divided into two categories of cerebral infarction and transient ischemic attack (TIA). Based on the clinical findings and its related basic research results, the following points can be briefly summed up: firstly, the causes of cerebral infarction are due to vascular occlusion (embolus) and the role of hypoperfusion is in the second place. The role of hypoperfusion in the causes of cerebral infarction might be mediated by the microemboli. The occurrence of cerebral infarction is nothing more than the following situations: (1) extracranial to intracranial arterial thrombosis; (2) intracranial artery to its branch artery immobilization; (3) intracranial arterial thrombosis in situ; (4) atherosclerosis occlusion in the end of the intracranial artery or cardiogenic emboli; (5) other factors resulting in the formation of emboli or arterial dissection or occlusive vascular inflammatory. Therefore, we speculate that if there are no emboli (dynamic emboli or emboli in situ), there might be no cerebral infarction. Secondly, according to the condition of vascular stenosis and arterial intimal lesions, the vessel background of TIA can be divided into four categories as follows: (1) with large vessels with stenosis and arterial intimal vulnerable lesions; (2) without vascular stenosis and arterial intimal vulnerable lesions; (3) with large vessels with stenosis but without vulnerable artery intimal lesion; (4) without large vessels stenosis but with arterial intimal vulnerable lesions. Based on the different background of the vessel (the vulnerability of vessels), their cause varies from hypoperfusion to microcephalic induced by hypoperfusion. In this paper, we review the studies on the etiologies of ICVD and give some proposal and viewpoints on the correlation between embolism and hypoperfusion.

## 2. Is the Mechanism of Cerebral Infarction Vascular Stenosis Induced Hypoperfusion or Unstable Plaque Falling off due to Embolism?

**Figure 1:**
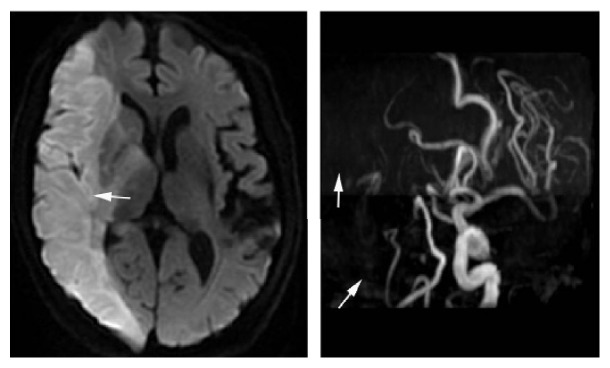
This figure showed images of infarction with artery to artery embolism which resulted from unstable atherosclerotic plaque falling off or in situ emboli formation.

The hypoperfusion has been considered as another important cause of ischemic stroke beside embolism. Since the middle of last century, most of the cerebral infarction was attributed to blood flow reduction or interruption resulting from intracranial and extracranial artery stenosis or occlusion and their arteriosclerosis. In all circumstance, there is no doubt that oxygen-glucose deprivation will lead to the brain tissue death. There is indeed no controversy for small artery occlusion and embolic material occlusion in intracranial branch resulting in cerebral infarction. However, in case of severe stenosis existing in a large vessel such as severe stenosis of the internal carotid artery or cerebral artery, is it hypoperfusion or unstable plaque which results in embolism or hypoperfusion-mediated microemboli formation based on stenosis? These problems still remain debatable up till now. It was previously known that intracranial and extracranial artery stenosis was a major risk factor of cerebral infarction, especially there is intracranial arteries stenosis found in vascular assessment: asymptomatic stenosis > 80%, symptomatic (TIA or stroke attack) stenosis > 50%, or degree of stenosis < 50%, but there is ulcerative plaque; interventional treatment is always performed to prevent possible ischemic stroke. However, it was found that a considerable part of patients with intracranial and extracranial stenosis, especially patients with extracranial stenosis, has no history of stroke in general in clinical practice. A prospective study [1] reported that stroke recurrence in patients with symptomatic middle cerebral artery (MCA) stenosis is much higher than that in patients with asymptomatic MCA stenosis, which seemed to indicate the inner correlation between MCA stenosis and the risk for stroke recurrence (in fact, the conclusion is not universal; we should not only focus on these findings; more importantly, their background and the prerequisite of this conclusion should be paid more attention to), but the premise of this correlation is that the patients had ever experienced cerebral ischemic events. (Depend on whether there are ischemic events or not; the vascular background between the two groups is completely different.) This study enrolled 102 patients with MCA occlusion or stenosis. The rate of ischemic events in 56 patients with asymptomatic MCA stenosis is only 2.8% during a mean follow-up of 31 months. Other similar studies also showed that the incidence of stroke in patients with asymptomatic intracranial vascular stenosis is low, generally in the range of 3 to 29.6% [2]. In the North American Symptomatic Carotid Endarterectomy Trial (NASCET), there was strong correlation between the severity of stenosis and stroke risk. However, this relationship becomes less clear in asymptomatic patients [3]. Even for the patients with the same background of intracranial vascular stenosis, vascular stenosis factors have different effect on the incidence of stroke. Why different incidences of stroke under the same premise of vascular stenosis background exist? It is clear that this difference lies in the intimal lesions of stenosis artery. That is to say, whether there exists vulnerable lesion in narrow arteries is the principal factor which determines the occurrence of stroke. Recent study suggests that change patterns of intracranial vessel wall described by 7.0 tesla (T) MRI are related to different ischemic infarcts types and etiologies [4]. Similarly, for patients with intracranial vascular stenosis and a history of cerebral ischemic events (namely, symptomatic patients with intracranial vascular stenosis), there are arterial intima vulnerable atheromatous lesions in their arteries, consequently the original and objective conditions for embolus generating. In contrast, for asymptomatic patients with intracranial vascular stenosis, the probability of the artery intima vulnerable lesions is very low. There is no objective condition causing the emboli generating and the incidence of cerebral ischemic events is naturally very low. The strong risk factors for cerebral infarction recurrence should be the vulnerable atheromatous lesions in artery intima. Mild to moderate arterial stenosis, inflammatory infiltration, and dyslipidemia are the main characteristic risk factors for unstable plaque. Other studies found that the incidence of cerebral infarction in patients with mild and moderate internal carotid artery stenosis is greater than in patients with severe internal carotid artery stenosis [5]. Mild stenosis always exists in types IV-V of unstable plaque (AHA plaque classification), which is prone to rupture leading to consequent occurrence of ischemic cerebrovascular events. Microemboli were the only effective factor which could predict stroke recurrence for patients with MCA stenosis. Previous study revealed that there were more high-risk vulnerable atherosclerotic plaques in these patients' vessels with vulnerable atheromatous lesions, even in patients with mild atherosclerosis [6]. These studies suggest that intracranial and extracranial vascular stenosis is not a dominant factor for cerebral infarction; the presence or absence of unstable plaque or the formation of microemboli based on stenosis is more important than stenosis degree. In patients with cerebral infarction combined with intracranial and extracranial stenosis, there are two kinds of pathogenesis of cerebral infarction as follows: firstly, instability plaque in stenosis vessels falls into the brain; in the second place, hemodynamic changes based on artery stenosis (hypoperfusion, abnormal changes in blood flow velocity, laminar flow change or destruction, swirl, or turbulence) induced the formation of microemboli (Figure 1). As long as there is no plaque falling off from extracranial to intracranial artery leading to embolization, it often only displays TIA rather than cerebral infarction. Of course, frequent TIA can progress to cerebral infarction, which is due to a large amount of microemboli formation and washing capacity of emboli decreases. Previous study demonstrated that microemboli from carotid artery could cause predominantly middle cerebral or ophthalmic artery territory TIA [7]. Microemboli signals (MESs) were detected significantly higher in patients with partial or total anterior circulation infarcts (39.1%) than in those with lacunar infarcts (26.0%) or TIA (27.3%) [8]. Most cerebral infarction which occurred in this case displays good prognosis. Therefore, existence of vulnerable plaque (plaque with uneven surface, uneven texture, or ulcers and intraplaque hemorrhage, the exposed collagen fibers on the injured endangium, which are likely to arouse platelet-activating) and arterial intima lesions is the principal factors for cerebral infarction. Plaque falling off or hemodynamic changes (or damage) induced microemboli formation, leading to embolism, is the principal mechanism of ischemic cerebrovascular events. The correlation between stenosis degree and MESs has been previously studied which led to somewhat controversial results. Some studies found that MESs were significantly more frequent in patients with higher degree of carotid stenosis [6, 9], while other studies did not find the association between the presence of MES and an increasing in carotid stenosis degree [10–13], which suggested there might be factors other than stenosis degree playing a major role in determining high-risk condition of ischemic events. Though there is intrinsical link between artery stenosis and atherosclerotic plaque. As it has been said above, unstable plaque always exists in the mild stenosis artery, which is a strong risk factor of ischemic stroke [14]. In contrast, most of the severe stenosis artery exists in stable plaque, and then the risk of infarction deceases. The role of the artery stenosis factor in ischemic stroke is much smaller than the unstable plaque [15]. Rupture of unstable plaque plays a leading role in the occurrence of ischemic stroke. Not only improving perfusion but also antiartery atherosclerosis and plaque stabilization should be performed in the artery stenosis for treatment. It can be proved by the ideal preventive effect of ischemic stroke obtained in the prevalence of carotid endarterectomy in western countries. Therefore, the fundamental mechanism underlying cerebral infarction is the rupture of unstable plaque. Recent study confirmed that the rate of microembolus positivity increased in patients with atherosclerotic thrombotic cerebral infarction and unstable plaques [16]. Hypoperfusion induced by artery stenosis might contribute to microemboli generating and block the responsible arteries. In a word, the core factor for cerebral infarction is emboli and its focal mechanism is occlusion.

## 3. The Pathogenesis of Watershed Cerebral Infarction Is Hypoperfusion

Up till now, the mechanism of watershed infarction still remains inconclusive. Hypoperfusion induced by artery stenosis is often regarded as the main mechanism of watershed infarction. Hypoperfusion and impairment in the washing capacity of emboli are usually bracketed together. A PET study on the patients with chronic internal carotid artery occlusion and without border zone infarct found that there was no difference in oxygen uptake rate between cortical and border zone tissues with contralateral and normal border zone, which prompted that internal carotid artery occlusion-mediated hypoperfusion did not make the oxygen uptake rate significantly increased in the weak blood flow border zone [17]. It was not as we previously thought that perfusion reduction resulted in the occlusion of the internal carotid artery, consequently leading to border zone infarction eventually. In fact, oxygen uptake in brain tissue increases under normal circumstances due to cerebral collateral circulation and autoregulation compensatory; then mere hypoperfusion was insufficient to directly lead to cell membrane pump failure and cerebral infarction. In fact, brain ischemia from mere hypoperfusion always only displayed TIA or syncope attacks in clinical practice, and cerebral infarction rarely occurred. For example, patients with severe aortic stenosis mostly present syncope attack (manifestation of typical systematic hypoperfusion). For the patients undergoing prolonged cardiac arrest who recovered from successful cardiopulmonary resuscitation, even if there existed severe hypoxic-ischemic encephalopathy, acute large area of cerebral infarction rarely occurred. Most of the patients with severe internal carotid artery stenosis rarely suffered severe stroke. The rate of stroke in patients with asymptomatic internal carotid artery occlusion is just 2%-3% per year. In addition, severe arterial occlusion due to acute carotid artery dissection usually results in TIA [18, 19]. These clinical evidences mentioned above indicate that the condition of mere hypoperfusion usually exists. Sometimes even if there is evidence of brain tissue damage (DWI) positive abnormal signal intensity. in general, the prognosis is good or presenting with reversible and ischemic defect of the nerve function (RIND). Moreover, mechanisms of watershed cerebral infarction (WCI) and TIA always overlap each other. Previous experiments or clinical studies had found that early ischemic preconditioning could increase tissue tolerance to ischemia and hypoxia, which would exert a protective role [20, 21]. In fact, hypoperfusion has a role of ischemic preconditioning in a sense. From the perspective of prognosis for tissue ischemia injury, hypoperfusion is beneficial rather than harmful. This is one of the reasons why patients with the WCI and cerebral infarction from TIA display good outcome.

Maybe there might exist a query. Generally speaking, patients with WCI always have large artery stenosis. However, why is hypoperfusion not the main cause of WCI? There is a relationship between microemboli formation with hypoperfusion, which should be worthy to be paid more attention to. In fact, there is an inner correlation between them. Large artery stenosis is the main factor which induced hypoperfusion and hemodynamic changes (impairment). In addition, systemic hemodynamic changes (various causes induced low blood pressure). It is vulnerable to form microemboli under the premise of hypoperfusion, hemodynamic changes, and vascular intima vulnerable lesions. Among these factors, the latter factor plays the prominent role in microemboli formation. Why does mere systemic hypotension always lead to syncope rather than cerebral infarction? Even though there is presence of systemic hypoperfusion or hemodynamic abnormalities, microemboli is not easy to form in the absence of arterial intima vulnerable lesions, which again prove that mere hypoperfusion generally does not easily lead to the cerebral infarction. This can also be interpreted as the reason why patients with severe aortic valve stenosis and internal carotid artery dissection always show TIA rather than cerebral infarction. From the perspective of treatment, antiplatelet aggregation therapy displays good effect for the WCI with vascular stenosis, which could reveal that microemboli formation might play the prominent role in the formation of WCI. Indeed, increasing blood volume and blood pressure also can improve the prognosis of WCI in the clinical practice. Improving perfusion and hemodynamics can eliminate or weakens the necessary condition for the microemboli formation, and at the same time, the washing ability of microemboli is enhanced. Previous autopsy studies indicated that visible platelet aggregation and leukocyte (white blood clots) could be found in the border zone of watershed infarction with or without large arteries stenosis [22]. Many cortical watershed infarctions were caused by microemboli, and perfusion around the cerebral infarction did not significantly decrease [23]. The degree of stenosis was involved in distal hemodynamic impairment in the territory of WCI, and the degree of plaque inflammation was related to large amounts of microemboli formation. Watershed infarcts resulted either from hemodynamic impairment secondary to severe vessel stenosis or from microembolism secondary to plaque inflammation. From the view of the background of this study, the patients were not stratified by vascular lesions background (stenosis and the arterial intima vulnerable lesions). We speculate that the arterial intima vulnerable lesion is a necessary condition for microemboli formation, and hypoperfusion and hemodynamic changes are the incentive or contributing factor of microemboli formation. In the case of hypoperfusion and hemodynamic impairment, microemboli are too difficult to be washed out and are prone to form in the border zone (distal). It is so called “microemboli elimination disorder,” which is the key node among hypoperfusion, cerebral embolism, and cerebral infarction. Therefore, as long as cerebral infarction occurred, its main cause is occlusion of the responsible artery by embolus. The WCI, commonly referred to as border zone infarcts, includes posterior external watershed infarction ((a cortical border zone infarct at the junction of the territories of the posterior cerebral artery (PCA) and MCA), anterior external watershed infarction (a cortical border zone infarct at the junctions of territories of the anterior cerebral artery (ACA) and MCA)), and internal (subcortical) watershed infarction ((subcortical border zone infarcts at the junctions of territories of the cortical and deep perforating branches of MCA)). In fact, external watershed infarction can be considered as the cortical watershed infarction or cortical infarction. Whether hypoperfusion-mediated microembolus could lead to cortical infarction, the answer is yes. In this sense, the external watershed infarction is usually caused by emboli from the proximal end of the large arteries leading to cortical branch embolization. At the same time, hypoperfusion in the cortical branch or poor circulation makes the washing ability of microemboli impaired. In general, there are lots of anastomosis between the gyri in the cerebral cortex, enough to make microemboli removed before the formation of large ischemic damage or compensated by rich collateral circulation. If substantial amount of microemboli exceeds the brain tissue's capacity threshold of microemboli elimination, then cortical infarction may occur. If the cerebrovascular autoregulation or collateral circulation in the peripheral (cortical) and internal (subcortical) watershed border zone is weak, the cortical and subcortical infarctions would occur simultaneously when being combined with large vascular stenosis. Internal border zone infarction only occurs under the condition of small amount of microemboli. (The location of the watershed infarction is linked to the responsibility arteries undergoing hypoperfusion.) If the collateral circulation of border zone is abundant, only cortical border zone infarction occurs under the condition of large amount of microemboli (due to the selective pressure of the laminar flow of the blood, most of the microemboli enter into the cortex) or is only showing TIA. Numerous studies found that microinfarcts preferentially occurred in cortical or subcortical regions [24, 25]. Cerebral infarction in DWI displays small and dense lesions; especially the cortical lesions more possess these characteristics. Lesions in border zone infarcts can be integrated into the sheet due to inadequate collateral circulation and large amounts of microemboli. Whether lesions of watershed infarction are integrated or not depends on the condition of collateral circulation and the amount of microembolus.

**Figure 2:**
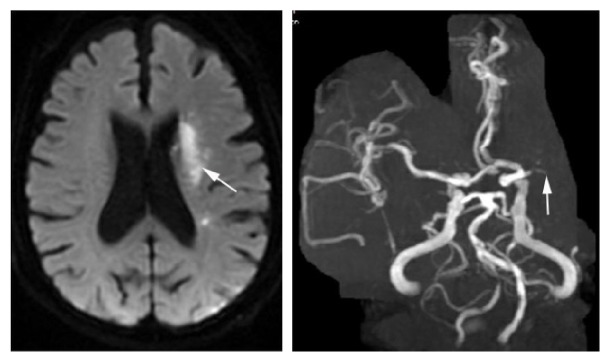
This figure showed the images of internal border zone infarction under the condition of hypoperfusion induced by severe vessel stenosis. Angiography of MRA revealed the occlusion of the left middle cerebral artery.

**Figure 3:**
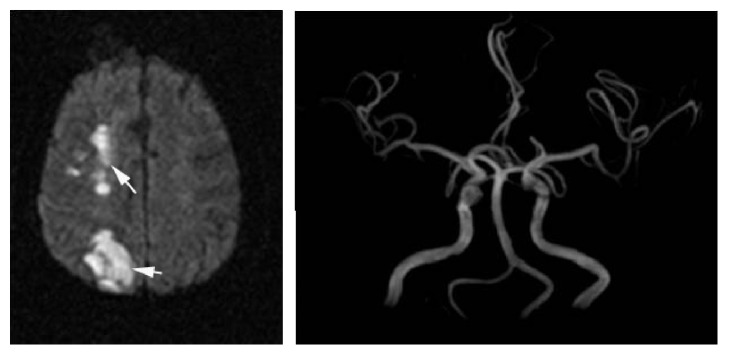
This figure showed the images of cortical watershed infarction induced by embolization. Angiography of MRA revealed that there was no occlusion of the right middle cerebral artery.

Internal border zone infarction mainly results from hemodynamic impairment secondary to severe lumen stenosis, while cortical watershed infarction is due to microembolism which is secondary to inflammation or fragmentation of plaque. The results showed that there were different mechanisms between the internal and cortical border zone infarction. However, we analyze the relevant research background and then find that the core mechanism does not yet go beyond the microemboli level. The key problem lies in the different source where microemboli come from. For internal watershed infarction (subcortical), microemboli originate from the supply arteries in the internal border zone; that is to say, the microemboli spontaneously form within the arteries (mostly in the distal secondary branches of the narrow arteries) which supply the internal border zone under the condition of hypoperfusion induced by severe vessel stenosis (Figure 2). For cortical watershed infarction, microemboli almost originate from the proximal end of the artery or heart. Most of the microemboli enter into the cortex due to the selective pressure of the laminar flow of blood. When the washing ability of microemboli decreases, cortical watershed infarction would be not difficult to occur (Figure 3). Therefore, the cause of cortical watershed infarction was attributed to embolization. This can explain that different types of watershed infarction have different vascular backgrounds.

**Figure 4:**
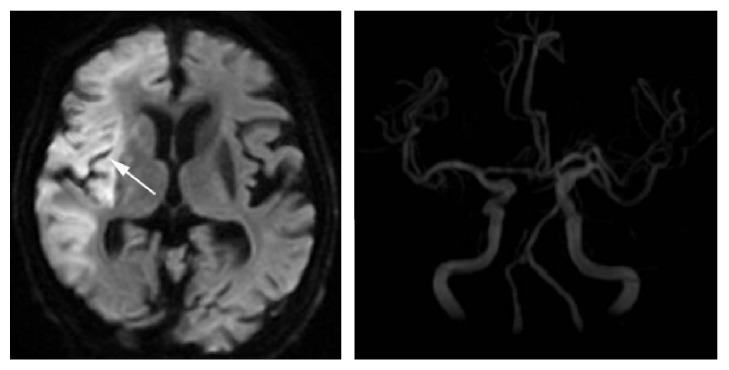
This figure showed the images of infarction with cardiogenic embolism induced by embolization. Angiography of MRA revealed that there was no occlusion of the right middle cerebral artery. The emboli might go to pieces during the process of their migration. The cortical infarcts display multiple ischemic lesions in MRI imaging, which is usually larger and more scattered than that caused by hypoperfusion.

Another case is that multiple cortical infarctions occur under the condition of being without significant stenosis or only with artery atherosclerosis. Because there is no inducement of hypoperfusion, artery to artery embolization (or cardiogenic embolism when there exists atrial fibrillation or atrial myxoma) should be responsible for this case. If emboli go to pieces during the process of their migration, the cortical infarcts display multiple ischemic lesions in MRI imaging, which is usually lager and more scattered than that caused by hypoperfusion. If microemboli are larger and not easily smashed to pieces, the lesions are prone to aggregation and fusion (blocking the parent artery of cortical perforating branches) (Figure 4), which is significantly different from the MRI imaging pattern of cortical watershed infarction (external border zone infarction) caused by microemboli induced by hypoperfusion.

**Figure 5:**
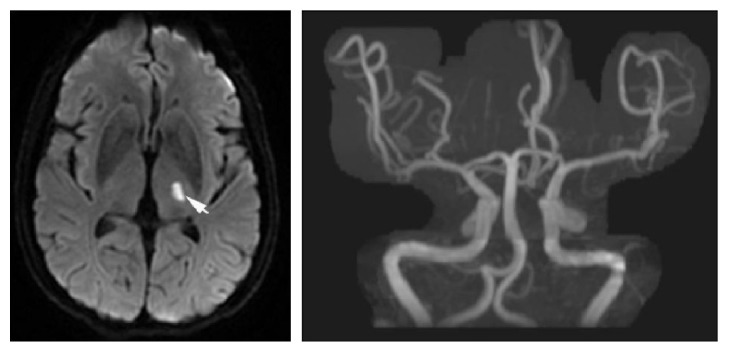
This figure showed images of symptomatic lacunar ischemic stroke which belongs to occlusion of small perforating branch. Angiography of MRA revealed that there was no occlusion of a cerebral artery.

Another type of infarction is symptomatic lacunar ischemic stroke which belongs to cerebral small vessel disease. Recent study reported that it (account for between 20% and 30% of all brain infarctions [26]) resulted from occlusion of a single penetrating artery by microatheromas or lipohyalinosis and rarely from an intracranial atheromatous branch disease (Figure 5). The addition of clopidogrel to aspirin not only did not reduce significantly the risk of recurrent lacunar stroke, but also increased significantly the likelihood of hemorrhage and fatal outcome [27]. Previous study of nonhuman primates showed that the direct injection of emboli into the internal carotid artery resulted in <6% of deep brain infarcts. Similarly, a proximal embolic source may be present in at least 10% of symptomatic deep brain infarcts, which suggests that intrinsic disease is a likely mechanism, and a proximal embolic source cannot be completely excluded [26]. It suggested that lacunar infarction might be not primarily nonatherothromboembolic, and secondary prevention aimed at preventing atheroma progression seems not be effective.

Patients with ischemic stroke of unusual cause (ISUC) presented a lower in-hospital mortality rate (7.1% versus 14.4%; *P* < 0.05), were more frequently symptom free at discharge (35.7% versus 25.80%; *P* < 0.05), and experienced a longer mean length of hospital stay (23.7 days versus 18.2 days; *P* = 0.06) than non-ISUC patients. The relevant studies suggested that ISUC was infrequent, etiologies were numerous, and hematologic disorders were the most frequent cause [28]. Another study reported that cardioembolic infarctions are one of the subtypes of ischemic acute phase of stroke. The in-hospital mortality rate of cardioembolic infarction was 27.3% as compared with 0.8% for lacunar infarcts and 21.7% for atherothrombotic stroke [29].

## 4. TIA'S Mechanisms: Microemboli or Hypoperfusion?

For the mechanisms of TIA, there still remain several hypotheses including microemboli, hemodynamic impairment, vasospasm, abnormal blood components, and arterial tortuosity variation. In clinical practice, based on whether there exist vascular stenosis and arterial intima vulnerable lesions, four conditions might exist as follows: (1) being combined with large artery stenosis and vulnerable artery intima lesions; (2) being without any large artery stenosis and vulnerable artery intima lesions; (3) being with large artery stenosis but without vulnerable artery intima lesions; (4) being without large artery stenosis, but with vulnerable artery intima lesions. Under the different backgrounds of vessels and arterial intimal lesions, different etiologies vary from hypoperfusion and hypoperfusion induced microemboli.

Firstly, for TIA patients with large artery and artery intima vulnerable lesions, especially for the patients who repeatedly experienced stereotypical attack, its cause can be regarded as hypoperfusion-mediated microemboli formation which plays the dominant role. Under the condition of hypoperfusion, microemboli are not difficult to form in the arteries with intima vulnerable plaque and the hemodynamic instability [30]. The location where the microemboli forms mainly lies in the distal point of stenosis artery. The microemboli essentially fixedly enter into the same intermediate vessels under the selective pressure of fluid mechanics. Then the clinical manifestations of such type of TIA are always rigidly fixed. If the border zone or collateral circulation of the remote cortical branch is still normal, or the formation of microemboli is less, which does not go beyond the threshold of brain tissue scavenging microemboli, it can only represent with TIA. These types of TIA depend on the brain tissue hypoxia tolerance in the hypoperfusion area, the condition of stenotic artery, and blood supply of responsible arteries. Hemodynamic disorders are prone to happen because of large artery stenosis. On the one hand, hemodynamic disorders provide conditions for the formation of microemboli, and on the other hand, it can make embolectomy reduce the washing ability of microemboli. Consequently the formed microemboli could not be easily washed out. Therefore, this condition can easily progress to cerebral infarction. Times for development from TIA to cerebral infarction are relatively little. That is to say, TIA always happens once or a few times and then would progress to cerebral infarction. Once cerebral infarction occurs, it can easily deteriorate to progressive cerebral infarction. Under such background of vessels, core factor of cerebral infarction, emboli, is not difficult to form and makes the washing ability of emboli impaired. In the case of arterial intima lesions, the in situ thrombosis is more prone to form, which always makes the condition deteriorated. Approximately, 1/3 of the patients with TIA could progress to cerebral infarction in clinical practice. For patients with TIA, the primary management is to improve the hemodynamics and perform the antiplatelet aggregation therapy and, at the same time, to rapidly complete vascular assessment in order to provide evidences for the next clinical decision and assessment for stroke risks. Secondly, for TIA without large artery disease and artery intima vulnerable lesions, its main mechanism is hypoperfusion. Such type of TIA can be divided into two subtypes: (1) systemic hypotension induced hemodynamic changes (autonomic reflex always involves in this process), mainly presenting syncope or drop attacks (belonging to posterior circulation, due to hypoxic-ischemic tolerance in posterior circulation dominated areas is relative poor); (2) penetrating artery hypoperfusion (belonging to small vessel diseases which differ from the mechanism of macrovascular diseases); hemodynamic changes lead to perforating artery hypoperfusion; consequently the corresponding brain tissue undergoes ischemia, which might be accompanied by the local small amount of microemboli formation. There is not any effective method to detect the abnormality of perforating artery. It should be noted that small perforating arteries can lead to signs and symptoms similar to macrovascular disease (unilateral limb weakness or speech impairment, partial body sensory disorders, dizziness, nausea and vomiting, bulbar paralysis, and other symptoms and signs, this is so called “small vessels disease leading to large infarction”). Such TIA always has accidental external stimulation factors, so the patients obtain long-term remission after they experience TIA only once or a few times (1/3 of patients with TIA). Third, for the TIA with large artery stenosis but without artery intima vulnerable lesions, its principal cause is hypoperfusion. Under the premise of hemodynamic changes or damage, the area of brain tissue supplied by the narrow or compensatory vessel undergoes a short-term hypoperfusion and hypoxia-ischemia, consequently resulting in focal neurological defects. For timely and effective supplementary blood volume, perfusion improvement is the best choice of treatment.

In addition, this circumstance has a relatively good outcome, even if progressing to cerebral infarction, which mostly shows watershed infarction. For experiencing TIA (ischemic preconditioning), cerebral infarction is always not serious and the prognosis is always fair in nonlacunar ischemic strokes; thus suggesting that TIA possibly displays a neuroprotective effect by inducing ischemic tolerance [31]. It may explain why the clinical prognosis of patients with cerebral infarction from TIA always varies from person to person. Fourth, for TIA without large artery stenosis, but with artery intima vulnerable lesions, its main cause is microemboli formation. There exists a systemic factor for hypoperfusion, and microemboli produce under the premise of artery intima vulnerable lesions. Amount of microemboli does not exceed the threshold of washing out the microemboli in brain tissue, which always leads to TIA rather than infarction. In addition, due to not combining with large artery stenosis, there is generally no significant hemodynamic compromise (excluding systemic hypotension factor which is different from the focal hypoperfusion mentioned above). Washing capacity of emboli is full even if there exist microemboli. The clinical manifestations always show TIA recurrence, which may help explain why part of TIA patients (about 1/3) could undergo long-term and multiple episodes without deteriorating to cerebral infarction.

**Figure 6:**
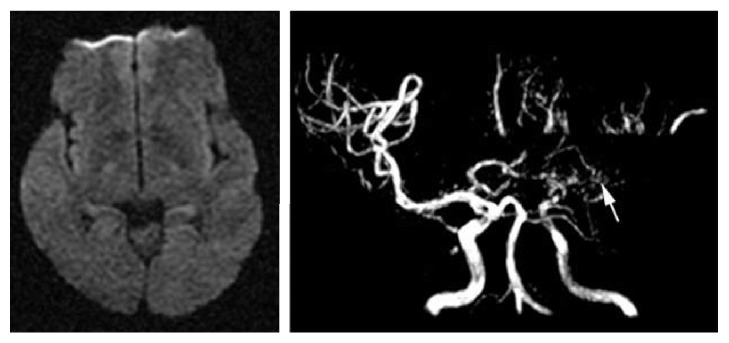
This figure showed images of limb shaking syndrome (LSS) combined with moyamoya disease.

**Figure 7:**
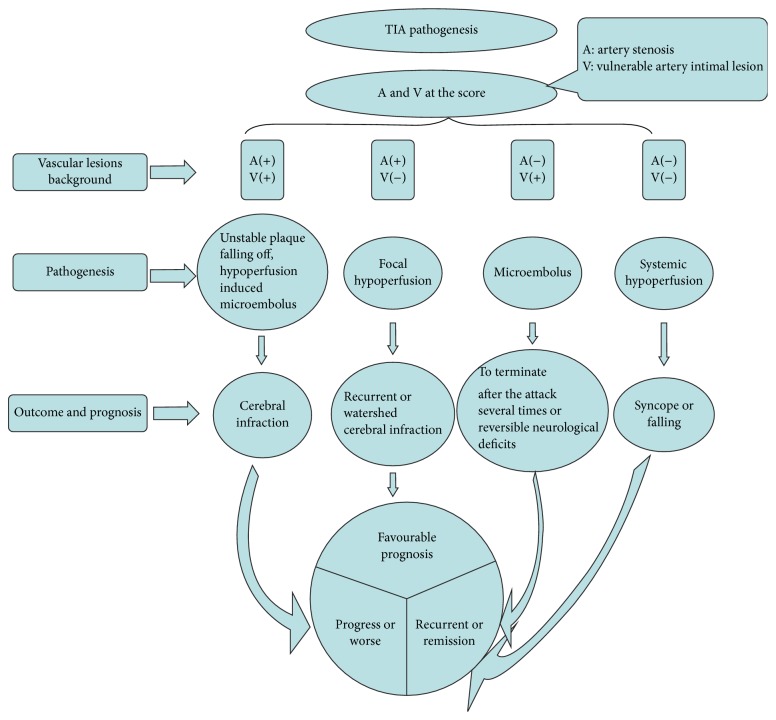
This figure shows the relevant etiologies of TIA and its corresponding different outcomes.

Clinically, there is a particular type of TIA, limb shaking syndrome (LSS). It is a very effective method to explore its mechanism through this particular type of TIA (it can be regarded as an ideal model of hypoperfusion). Such type of TIA is rare and more commonly observed in younger patients and rarely in elderly patients. These patients are generally combined with intracranial and extracranial artery stenosis or moyamoya disease, especially in the MCA secondly to intracranial internal carotid artery stenosis (Figure 6). Clinical onset form is more rigid, which is characterized by fixed limb shaking as the main clinical manifestations. Some represent dancing-like action. Time duration of an attack is generally not more than half an hour and is mostly relieved in a few minutes to 10 minutes. Increasing blood volume and vasodilator use are effective treatments for this type of TIA caused by hypoperfusion. Previous case-control studies also suggested that there were evidences of hemodynamic impairment in the LSS TIA with internal carotid artery occlusion [32]. TIA patients with artery stenosis and intimal vulnerable lesions are easy to deteriorate to cerebral infarction. These clinical evidences also confirm the fact that how hypoperfusion and microemboli involved in the mechanisms of TIA might depend on artery stenosis and arterial intimal lesions. Other risk factors such as aneurysm, arterial inflammation, and abnormal blood components (such as polycythemia vera, thrombocytopenia, or polycythemia, and anticardiolipin antibody syndrome), cardiogenic emboli (atrial mural thrombus, mitral valve vegetation, atrial septal abnormalities such as atrial septal aneurysm, patent foramen ovale atrial septal defect), and cholesterol crystals all take microemboli as the final pathway to the development of TIA. Although there are a large number of risk factors for TIA, regarding the larger artery stenosis and arterial intimal lesions (vessel wall abnormalities) as the main line, the mechanisms of TIA do not go beyond the range of hypoperfusion and microemboli, namely, hypoperfusion or hypoperfusion induced microemboli formation or microemboli, which also provide a better understanding for the internal causes underlying these three distinct outcomes and prognosis of TIA (Figure 7).

## 5. Conclusion

**Figure 8:**
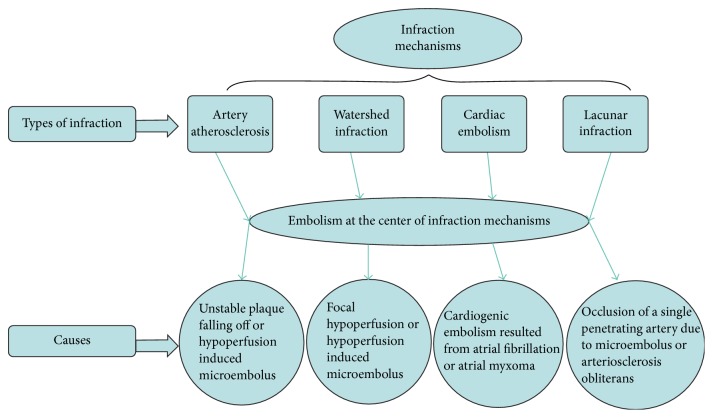
This figure showed the etiologies of infarction, and emboli lie in the center of cerebral infarction mechanisms.

In summary, this paper proposed several different opinions on the mechanisms of ICVD: first, the vulnerability of the arterial intima (such as endangium ulcers, unstable plaque) is the core risk factor for cerebral infarction. Embolism caused by microemboli formation induced by unstable plaque or artery stenosis induced hemodynamic compromise is the main cause of cerebral infarction. The role of stenosis factor in ischemic stroke is much smaller than the unstable plaque as well as other artery intima vulnerable lesions. Secondly, core etiology for watershed infarction and subcortical infarction is still embolization secondary to hypoperfusion induced by the large artery stenosis. Emboli formation induced by a variety of factors mainly resulted from the occlusion of responsible vessels and is the core mechanism of cerebral infarction (Figure 8). Thirdly, there is a correlation between embolism and hypoperfusion, and different causes of ICVD vary from hypoperfusion and hypoperfusion induced microemboli depending on the vulnerability of vessels. This inference might enrich and provide better understandings of the underlying cause of ischemic cerebrovascular events in the traditional sense.
